# Effects of different acupuncture strategies on the prognosis and inflammatory factor levels associated with the treatment of knee osteoarthritis

**DOI:** 10.1016/j.clinsp.2026.100871

**Published:** 2026-02-14

**Authors:** Jun Li, Di Wu, Xueli Chen, Guanhua Li

**Affiliations:** a Department of Rehabilitation Medicine, Jiujiang City Key Laboratory of Cell Therapy, JiuJiang NO.1 People's Hospital, Jiujiang, China; b Department of Rheumatology and Immunology, Jiujiang City Key Laboratory of Cell Therapy, JiuJiang NO.1 People's Hospital, Jiujiang, China; c Department of Clinical Laboratory, Jiujiang City Key Laboratory of Cell Therapy, JiuJiang NO.1 People's Hospital, Jiujiang, China

**Keywords:** Acupuncture, Electroacupuncture, Inflammation, Knee osteoarthritis, SF-36 assessment form, WOMAC assessment form

## Abstract

**Background:**

Knee osteoarthritis is a degenerative joint condition characterized by the progressive degeneration of knee cartilage. Acupuncture therapy, as an alternative treatment, has shown significant efficacy in managing joint disorders. In the current study, the effects of four acupuncture strategies in treating knee osteoarthritis were compared and analyzed.

**Methods:**

123 cases diagnosed with knee osteoarthritis were included in the current study at the First People's Hospital of Jiujiang City from May 2022 to May 2023. The patients received different acupuncture strategies, including traditional acupuncture, electroacupuncture, warm acupuncture, and fire acupuncture. Then the information regarding the treatment effects on physical function and inflammatory cytokines was collected and analyzed.

**Results:**

The data showed that all four treatment strategies could improve the life quality of the patients, but the effects of electroacupuncture were stronger. The effects of warm acupuncture and fire acupuncture were weaker than electroacupuncture, but were stronger than traditional acupuncture. Regarding the effects on knee physical function and inflammatory indicators, electroacupuncture also showed better treatment effects than the other three treatments. The overall efficacy in treating knee osteoarthritis was comparable between different treating strategies, even though the effects of different acupuncture strategies on detailed items might be different.

**Conclusions:**

Collectively, the current study demonstrated that all the acupuncture strategies could improve the physical function and reduce pain in the knee, thereby improving the life quality of patients, with electroacupuncture showing the strongest effects on certain detecting items.

## Highlights

• Acupuncture could improve the physical function and reduce pain of knee.
• Acupuncture improving the life quality of patients.
• Electroacupuncture showed the strongest effects.

## Introduction

Knee osteoarthritis is a prevalent joint condition, often referred to as degenerative knee arthritis, which is characterized by the progressive degeneration of knee cartilage, leading to joint swelling, pain, and restricted movement.^1^^,^^2^ The pathology of the disorder involves a reduction in chondrocytes, dissolution of the cartilage matrix, and eventual loss of cartilage, resulting in the destruction of the articular cartilage tissue.^3^^,^^4^ Additionally, knee osteoarthritis is marked by bone hyperplasia, cartilage degeneration, the formation of bone spurs, and the occurrence of synovitis in the knee joint.^4^ Recent reviews have emphasized that knee osteoarthritis should not be considered only a “cartilage disease”, but rather a disorder of the entire joint. Pathological changes extend beyond cartilage to involve the subchondral bone, synovium, menisci, ligaments, periarticular muscles, and fat pads. These tissues collectively contribute to pain, stiffness, and functional decline, highlighting knee osteoarthritis as a complex, whole-joint failure rather than a single-tissue pathology.^5^, ^6^, ^7^ With economic development and improved living conditions, the average life expectancy has increased, and more people are participating in outdoor sports. Consequently, the proportion of individuals experiencing knee pain has risen significantly. Many retirees are now engaging in daily cultural and sports activities. Factors such as traffic accidents and other incidents have also contributed to the sharp increase in the number of patients with various types of knee arthritis.^6^ Currently, knee osteoarthritis primarily affects middle-aged and elderly individuals and can significantly impair the quality of life of patients and cast a great burden to public health system.

The treatment of knee osteoarthritis can be divided into two strategies: conservative and surgical. Conservative treatment primarily involves drug therapy such as oral Non-Steroidal Anti-Inflammatory Drugs (NSAIDs) for pain relief,^8^ glucosamine supplements for chondrocyte nutrition,^9^ and intra-articular injections of sodium hyaluronate to slow cartilage damage.^10^ Surgical treatments mainly consist of cartilage grafting and artificial joint replacement.^6^ In recent years, the development of minimally invasive techniques, such as knee joint exploration and debridement, has also been incorporated into clinical practice.^11^ However, the long-term effects of these minimally invasive procedures are often not ideal and can be costly. Therefore, treating strategies with less damages on knee and mild side effects are currently promptly explored to improve the quality of life of the patients.

In traditional Chinese medicine, knee osteoarthritis is categorized under bone thinness and bone atrophy. Generally, it is believed that paralysis is often caused by a combination of “wind”, “cold”, and “dampness”. However, based on current clinical observations of patients’ age, symptoms, signs, and imaging results, the etiology and pathogenesis of knee osteoarthritis are largely associated with deficiencies in the kidneys, liver, and spleen in the elderly. Additionally, it is also considered as a type of pain arising from blocked meridians and joints.^12^ Recently, the increased emphasis on health preservation by Traditional Chinese Medicine (TCM) has made the therapeutic advantages of TCM in treating knee joint inflammation become more widely recognized by patients. The use of traditional Chinese methods, such as herbal medicine, acupuncture, and massage, has gained widespread acceptance in handling bone disorders such as knee osteoarthritis. Research on TCM with animal experiments highlights that key cytokines like IL-1, TNF-a, serotonin, and prostaglandin E2 are identified as important mediators in the pathological process of knee osteoarthritis, with their tissue levels closely linked to inflammation development.^13^^,^^14^ Of these treating strategies, acupuncture therapy, as a complementary and alternative treatment, has demonstrated significant efficacy in managing Rheumatoid Arthritis (RA).^15^ It is particularly effective in improving clinical symptoms, slowing disease progression, and alleviating pain, with minimal adverse reactions and low cost. Acupuncture encompasses various techniques with distinct effects, and previous studies have shown that conventional acupuncture can enhance joint function and quality of life in patients compared to modern medicines. Currently, acupuncture treatment can be performed as traditional acupuncture, electrical acupuncture, warm acupuncture, and fire acupuncture in a clinic, and different studies have already shown their efficacy in handling joint disorders. However, no comparison between the three acupuncture types in treating knee osteoarthritis has been performed. Therefore, the current study collected the information of 123 knee osteoarthritis patients in our hospital. The treating outcomes of three acupuncture strategies were compared against different parameters, such as pain index and inflammatory cytokines associated with knee osteoarthritis. The data of the current study aimed to facilitate the selection of a proper acupuncture strategy in the clinical handing knee osteoarthritis, as well as providing additional information on the underlying mechanisms responsible for the therapeutic effects of acupuncture.

## Methods

### Patients

**Fig. 1:**
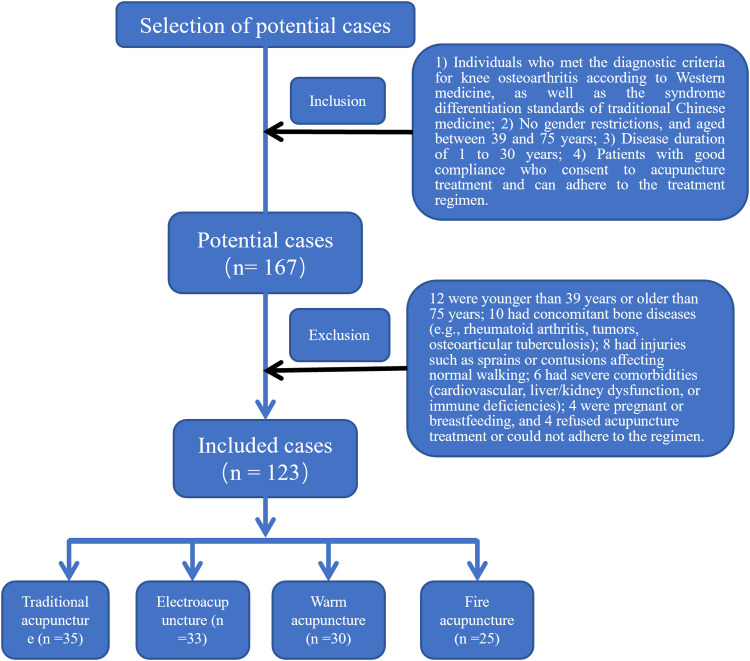
Selection flowchart of all the cases.

The current analysis enrolled 123 cases that were diagnosed with knee osteoarthritis at the First People's Hospital of Jiujiang City from May 2022 to May 2023 (Fig. 1). All patients were required to voluntarily agree to the use of their clinicopathological information in future analyses, such as in the current study and gave a written informed consent. The current study has been approved by the Ethics Committee of our institute, and was performed in accordance with the Declaration of Helsinki and STROBE Statement. The patients were divided into four groups based on the acupuncture treatment strategies they received. Treatment Group I: 35 patients were treated with traditional acupuncture; Treatment Group II: 33 patients were treated with electrical acupuncture; Treatment Group III: 30 patients were treated with warm acupuncture. Treatment Group IV: 25 patients were treated with warm acupuncture. For inclusion, cases should meet the following criteria: 1) Individuals who met the diagnostic criteria for knee osteoarthritis according to Western medicine, as well as the syndrome differentiation standards of traditional Chinese medicine; 2) No gender restrictions, and aged between 39 and 75 years; 3) Disease duration of 1 to 30 years; 4) Patients with good compliance who consent to acupuncture treatment and can adhere to the treatment regimen. Patients with following characteristics were excluded from the analyses: 1) Individuals who do not meet the aforementioned inclusion criteria, specifically those younger than 39-years or older than 75-years; 2) Patients with other bone diseases such as knee joint osteoarticular tuberculosis, tumors, rheumatism, and rheumatoid arthritis, which are not classified as osteoarthritis; 3) Individuals with concomitant sprains, contusions, or other injuries; 4) Patients with foot deformities, pain, or other conditions affecting normal walking; 5) Individuals with infections on the local skin where the treatment is to be applied or with deep swelling; 6) Patients with severe cardiovascular diseases, liver or kidney dysfunction, immune deficiencies, mental disorders, diabetes, or hematological diseases; 7) Pregnant or breastfeeding women; 8) Individuals receiving other treatments during the treatment period that may interfere with the efficacy observation.

### Treating strategies

Acupoints were selected based on the national higher education textbook “Acupuncture Therapeutics” used in traditional Chinese medicine institutions.^16^ The main acupoints were as follows: Ashi, Xiyan, Liangqiu, Yanglingquan. Supplementary acupoints were selected according to syndrome differentiation: Xuanzhong was used for kidney deficiency and marrow depletion Type; Xuehai was used for blood stasis and blocked collaterals type; Zusanli was used for yang deficiency and cold congealing type. Each acupuncture strategy was administered twice a week, and one course of treatment lasted for six weeks. The treatment efficacy was evaluated at the end of each course.

After acupuncture, patients were instructed to perform functional exercises for the knee joint, such as flexion and extension movements of the knee. These exercises were to be done three times a day, with each session consisting of ten repetitions, to improve the range of motion of the knee joint and strengthen the quadriceps muscle. The treatment of different acupuncture strategies was performed as described below.

### Traditional group

Patients were positioned either supine or seated. Ashi points were selected as local acupoints, with the most painful points used as acupoints. After routine disinfection, disposable sterile acupuncture needles (Huahua brand, Suzhou Medical Supplies Factory) were inserted directly into Ashi points, Xiyan, Liangqiu, and Yanglingquan to a depth of 1‒1.5 cm.

### Electroacupuncture group

The acupuncture was first performed as in the Traditional group. An electroacupuncture device (Xinsheng brand, Model SDZ-II, Qingdao Xinsheng Industrial Co., Ltd.) was connected to the main and supplementary acupoints. A continuous dense wave was used at a frequency of 50 Hz for 30 min. The intensity was set to the patient's tolerance level. The needles were retained for 30 min, then changed to sparse-dense waves for another 30 min.

### Warm acupuncture group

The main and supplementary acupoints were selected as described above, and the moxa stick was cut to a length of about 2 cm. One end was punctured with a coarse, sharp instrument. The moxa stick was ignited with an alcohol swab, and the punctured end was placed on the needle handle. A layer of heat-insulating paper was applied over the acupoint to prevent burns. When the moxa stick burned out, another section was replaced, with a total of three sections burned per session.

### Fire acupuncture group

Except for the procedures described in the Traditional group. The needle was heated in the outer flame about 1 cm above the intended insertion point at an angle of approximately 30 degrees, heating the front and middle segments of the needle until they were red-hot. The needle was then rapidly inserted vertically into the selected acupoint. The length of the heated needle had to be greater than the depth of insertion. After needling, the puncture site was quickly covered with Jingxiutang Dit Da Wan Hua Oil.

### Treatment outcome assessment

The treatment outcomes of different acupuncture strategies were assessed using SF-36 quality of life assessment form, WOMAC assessment form, JOA score, and VAS score.

### Statistical analysis

The mean ± Standard Deviation (SD) was used to represent normally distributed data, whereas the median and interquartile range were used to represent non-normally distributed data. The Fisher's exact test or the Chi-Square test was employed to compare the variations in the counting data. The differences between continuous data were compared using the Student's *t*-test for parametric testing and the Mann-Whitney *U* test for nonparametric testing. The statistical analyses and graph plotting were conducted either using GraphPad Prism version 8.0.0 for Windows (GraphPad Software, San Diego, California USA, www.graphpad.com) or using *R* language version 4.2.2 with a significance level of 0.05.

## Results

### Baseline clinicopathological information

A total of 123 qualified subjects were included and randomly assigned to the traditional acupuncture, electroacupuncture group, warm acupuncture group, and fire acupuncture group. There were 52 males and 71 females, and specifically, the traditional acupuncture group contained 14 males, the electroacupuncture group contained 13 males, warm acupuncture group contained 14 males, and the fire acupuncture group contained 11 males. No significant difference was detected between different groups regarding sex proportion (Table 1; *p* = 0.06). The average ages of the four groups were 51.6 ± 8.4 years, 55.1 ± 4.9 years, 52.0 ± 6.9 years, and 55.7 ± 8.1 years, respectively. No significant difference was detected between different groups regarding the average age (Table 1; *p* = 0.92). Regarding disease course, the average disease courses were 80.5 ± 13.8, 77.7 ± 8.3, 81.4 ± 13.8, and 79.8 ± 11.2 months, respectively. No significant difference was detected between different groups regarding the disease course (Table 1; *p* = 0.50).

**Table 1:** Clinicopathological information before acupuncture treatment.

	Traditional acupuncture (*n* = 35)	Electroacupuncture (*n* = 33)	Warm acupuncture (*n* = 30)	Fire acupuncture (*n* = 25)	p-value
**Age (year)**	51.6 ± 8.4	55.1 ± 4.9	52.0 ± 6.9	55.7 ± 8.1	0.06
**Male, n (**%**)**	14 (40.0%)	13 (39.4%)	14 (46.7%)	11 (44.0%)	0.92
**Disease course (month)**	80.5 ± 13.8	77.7 ± 8.3	81.4 ± 13.8	79.8 ± 11.2	0.50

### Effects of different acupuncture treatments on life quality of patients

The quality of life of patients in different groups was assessed using SF-36 life quality of life assessment form. As shown in Table 2, no significant difference was detected regarding physical health, physical role functioning, bodily pain, general health, vitality, social functioning, emotional role functioning, and mental health (*p* > 0.05) before the treatments. After the treatment, the data showed that parameters including physical health, physical role functioning, bodily pain, vitality, and social functioning were improved by all the four treating strategies (Table 2; *p* < 0.05); regarding general health, only patients in electroacupuncture group showed significant improvements (Table 2; *p* < 0.05); regarding mental health, only patients in electroacupuncture group and warm acupuncture group showed significant improvements (Table 2; *p* < 0.05); regarding emotional role functioning, no treatment strategies showed improved effects. Overall, the electroacupuncture group and warm acupuncture group showed better improvements on patients’ life quality, followed by fire acupuncture group, and the traditional acupuncture group showed the weakest improving effects assessed by SF-36 life quality assessment form (Table 2).

**Table 2:** Effects of different acupuncture treatments on life quality of patients assessed by SF-36 form.

		Traditional acupuncture (*n* = 35)	Electroacupuncture (*n* = 33)	Warm acupuncture (*n* = 30)	Fire acupuncture (*n* = 25)
**Physical health**	Before	59.9 ± 10.7	58.9 ± 10.9	55.9 ± 11.23	58.7 ± 9.0
	After	67.7 ± 7.6^a^	74.0 ± 9.5^a^^,^^b^	71.5 ± 6.0^a^^,^^b^	68.9 ± 11.6^a^^,^^b^^,^^c^
**Physical role functioning**	Before	48.5 ± 7.0	49.8 ± 10.4	49.2 ± 8.9	45.6 ± 11.0
	After	53.9 ± 7.9^a^	60.2 ± 9.7^a^^,^^b^	58.0 ± 11.4^a^	54.6 ± 7.8^a^
**Bodily pain**	Before	55.1 ± 11.8	51.8 ± 8.8	49.4 ± 14.0	56.2 ± 9.6
	After	71.3 ± 11.8^a^	76.0 ± 13.1^a^	71.4 ± 7.2^a^	74.9 ± 6.3^a^
**General health**	Before	36.2 ± 9.5	33.2 ± 9.7	33.3 ± 8.3	32.4 ± 7.1
	After	34.3 ± 9.3	46.2 ± 11.6^a^^,^^b^	34.2 ± 8.4^c^	35.9 ± 7.8^c^
**Vitality**	Before	35.5 ± 7.3	40.0 ± 7.1	36.3 ± 12.9	38.0 ± 8.2
	After	45.9 ± 12.9^a^	49.8 ± 11.9^a^	48.1 ± 11.3^a^	47.8 ± 7.4^a^
**Social functioning**	Before	37.6 ± 10.9	35.5 ± 8.4	36.7 ± 11.5	36.5 ± 9.3
	After	44.8 ± 10.5^a^	46.0 ± 5.4^a^	45.0 ± 6.4^a^	45.4 ± 13.9^a^
**Emotional role functioning**	Before	34.1 ± 8.4	38.9 ± 6.1	41.1 ± 10.8	37.4 ± 8.6
	After	40.3 ± 10.4	40.1 ± 11.9	41.7 ± 8.1	41.4 ± 4.3
**Mental health**	Before	40.4 ± 7.7	39.9 ± 14.5	40.1 ± 11.9	40.2 ± 19.8
	After	40.3 ± 10.4	49.1 ± 8.1^a^^,^^b^	47.7 ± 8.5^a^	41.0 ± 4.3^c^

a *p* < 0.05 vs. Before.

b *p* < 0.05 vs. Traditional acupuncture.

c *p* < 0.05 vs. Electroacupuncture.

### Effects of different acupuncture treatments on knee physical function of patients

The effects of different treatment strategies on knee physical function were assessed by the WOMAC assessment form. Before the treatment, no significant difference was detected regarding the pain, stiffness, physiological function, and overall score between different groups (Table 3; *p* > 0.05). After the treatment, all the scores were decreased by the acupuncture (Table 3; *p* < 0.05). The effects were majorly observed regarding pain, stiffness, physiological function, and overall score in the electroacupuncture group and warm acupuncture group, and regarding pain, physiological function, and overall score in the fire acupuncture group. Regarding patients receiving traditional acupuncture treatment, the improving effects were only observed for pain management. The results were similar to the life quality assessment using the SF-36 life quality assessment form, with electroacupuncture showing the strongest improving effects, followed by warm acupuncture, fire acupuncture, and traditional acupuncture (Table 3).

**Table 3:** Effects of different acupuncture treatments on knee physical function of patients assessed by WOMAC assessment form.

		Traditional acupuncture (*n* = 35)	Electroacupuncture (*n* = 33)	Warm acupuncture (*n* = 30)	Fire acupuncture (*n* = 25)
**Pain**	Before	9.8 ± 4.3	10.4 ± 3.0	11.8 ± 5.0	9.1 ± 2.8
	After	5.8 ± 1.9^a^	4.0 ± 0.6^a^^,^^b^	6.3 ± 1.1^a^^,^^c^	4.3 ± 1.2^a^^,^^b^^,^^c^
**Stiffness**	Before	3.8 ± 1.1	5.4 ± 1.3	4.7 ± 1.5	3.8 ± 1.1
	After	3.4 ± 0.6	1.6 ± 0.8^a^^,^^b^	1.2 ± 0.7^a^^,^^b^	3.0 ± 0.5
**Physical function**	Before	28.1 ± 6.0	30.0 ± 8.2	28.2 ± 6.7	29.1 ± 10.4
	After	24.5 ± 4.1	18.0 ± 4.7^a^^,^^b^	21.6 ± 5.0^a^^,^^b^	23.4 ± 4.6^a^
**Synthesis score**	Before	44.0 ± 10.5	42.4 ± 8.4	41.6 ± 8.1	44.6 ± 9.3
	After	38.7 ± 4.2^a^	27.4 ± 8.7^a^^,^^b^	30.5 ± 9.0^a^^,^^b^	27.5 ± 5.4^a^^,^^b^

a *p* < 0.05 vs. Before.

b *p* < 0.05 vs. Traditional acupuncture.

c *p* < 0.05 vs. Electroacupuncture.

### Effects of different acupuncture treatments on VAS and JOA scores of patients

The VAS and JOA scores were also employed for assessing the outcomes of the treatments. As shown in Table 4, Table 5, no significant difference was detected regarding the two scores between different groups before the treatments (*p* > 0.05). After the treatments, VAS scores were decreased by the treatments, and the effects of traditional acupuncture treatment were weaker than the other three strategies (Table 4; *p* < 0.05). Regarding JOA scores, all the treatments improved the score in different aspects. For instance, fire acupuncture and warm acupuncture had better improving effects on walking than electroacupuncture (Table 5; *p* < 0.05), while fire acupuncture and electroacupuncture had better improving effects on buckling function than warm acupuncture (Table 5; *p* < 0.05). The detailed results of JOA assessment were shown in Table 5.

**Table 4:** Effects of different acupuncture treatments on VAS score of patients.

.		Traditional acupuncture (*n* = 35)	Electroacupuncture (*n* = 33)	Warm acupuncture (*n* = 30)	Fire acupuncture (*n* = 25)
**VAS**	Before	6.1 ± 1.1	5.3 ± 1.7	5.8 ± 1.7	5.4 ± 1.3
	After	3.7 ± 1.2^a^	2.7 ± 0.6^a^^,^^b^	3.2 ± 1.0^a^^,^^c^	2.6 ± 0.6^a^^,^^b^^,^^c^

a *p* < 0.05 vs. Before.

b *p* < 0.05 vs. Traditional acupuncture.

c *p* < 0.05 vs. Electroacupuncture.

**Table 5:** Effects of different acupuncture treatments on JOA of patients.

		Traditional acupuncture (*n* = 35)	Electroacupuncture (*n* = 33)	Warm acupuncture (*n* = 30)	Fire acupuncture (*n* = 25)
**Walking**	Before	19.4 ± 5.2	21.1 ± 4.3	22.1 ± 7.7	21.3 ± 3.9
	After	24.0 ± 3.2^a^	25.7 ± 4.4^a^	26.2 ± 4.3^a^	28.0 ± 6.7^a^
**Stairs**	Before	15.0 ± 5.8	16.0 ± 4.0	16.6 ± 3.5	16.4 ± 4.6
	After	15.2 ± 5.6	18.0 ± 5.8	17.4 ± 3.2	18x.2 ± 6.3
**Stretch**	Before	31.5 ± 6.6	28.7 ± 5.6	29.0 ± 6.7	27.7 ± 7.0
	After	10.4 ± 0.6^a^	6.6 ± 0.8^a^^,^^b^	8.2 ± 0.7^a^^,^^b^	8.0 ± 0.5^a^^,^^b^
**Swell**	Before	8.0 ± 2.5	7.0 ± 1.2	7.0 ± 2.2	8.0 ± 2.4
	After	8.8 ± 4.0	10.5 ± 5.6^a^^,^^b^	11.5 ± 5.2^a^^,^^b^	10.7 ± 5.3^a^^,^^b^
**Synthesis score**	Before	73.2 ± 11.3	72.6 ± 10.3	72.7 ± 9.8	73.0 ± 10.0
	After	86.4 ± 7.0^a^	84.3 ± 7.6^a^	89.8 ± 10.5^a^	88.3 ± 8.9^a^

a *p* < 0.05 vs. Before.

b *p* < 0.05 vs. Traditional acupuncture.

### Effects of different acupuncture treatments on inflammatory indicators of patients

The treatment effects of different acupuncture strategies on the inflammatory factors of patients were assessed. The analysis results showed no significant difference was detected before the treatment between different groups (Table 6). After the treatment, all the inflammatory indicators were improved by acupuncture, with electroacupuncture showing the strongest improving effects, followed by fire acupuncture (Table 6).

**Table 6:** Effects of different acupuncture treatments on inflammation of patients.

		Traditional acupuncture (*n* = 35)	Electroacupuncture (*n* = 33)	Warm acupuncture (*n* = 30)	Fire acupuncture (*n* = 25)
**IL-6 (ng/mL)**	Before	3.1 ± 1.0	3.9 ± 1.3	2.9 ± 1.0	3.5 ± 0.0.9
	After	1.0 ± 0.3^a^	0.6 ± 0.2^a^^,^^b^	0.8 ± 0.3^a^	0.9 ± 0.3^a^
**TNF-α (fmoL/mL)**	Before	45.1 ± 9.2	42.9 ± 11.3	46.4 ± 8.3	44.6 ± 10.0
	After	15.4 ± 3.8^a^	9.0 ± 6.0^a^^,^^b^	10.0 ± 4.2^a^^,^^b^	9.5 ± 3.4^a^^,^^b^
**CRP (mg/L)**	Before	17.3 ± 7.0	20.9 ± 13.5	25.0 ± 11.0	23.9 ± 11.6
	After	10.5 ± 6.3^a^	5.7 ± 3.3^a^^,^^b^	7.5 ± 4.2^a^^,^^b^	8.9 ± 4.8^a^^,^^c^

a *p* < 0.05 vs. Before.

b *p* < 0.05 vs. Traditional acupuncture.

c *p* < 0.05 vs. Electroacupuncture.

### Overall treatment outcomes of different acupuncture treatments

The overall treatment efficacy of the four groups was not statistically significant (*p* > 0.05). Specifically, the traditional acupuncture group had a cure and improvement rate of 54.6%, with a total effective rate of 71.3%; the electroacupuncture group had a cure and improvement rate of 59.3%, with a total effective rate of 74.8%; the warm acupuncture group had a cure and improvement rate of 61.5%, with a total effective rate of 76.3%; the cure and improvement rate for the fire acupuncture group was 62.6%, with a total effective rate of 76.2%. Statistical analysis indicated that there were no significant differences in the total effective rates among the four groups (*p* > 0.05), suggesting that although the effects of different acupuncture strategies on detail items might be different, the efficacy in treating knee osteoarthritis is comparable between different treatment strategies.

## Discussion

Knee osteoarthritis is a chronic degenerative disease with a complicated pathogenesis, which can be induced by multiple factors. The disorder always presents with symptoms such as knee swelling and pain, soreness, restricted movement, and exacerbation with fatigue.^17^ In severe cases, it can lead to joint deformity, significantly impacting daily life and work, thereby causing substantial physical, mental, and economic burdens on society, families, and patients. With the continuous deepening of understanding and research on this disease in Western medicine, it can be managed through oral and topical anti-inflammatory and analgesic drugs, intra-articular injections of sodium hyaluronate, and oral cartilage-protective agents to slow disease progression and maintain knee joint function.^18^ However, although oral medications provide quick relief, long-term use has significant side effects and many manifestations, including inhibited collagen matrix synthesis, which results in necessitating surgical intervention as a remedial measure. However, surgery carries substantial risks and high costs, and is not suitable for patients with significant organ damage, making it difficult for many patients to endure. Considering these factors, the public often turns to TCM for the treatment of knee osteoarthritis. Throughout the long history of traditional Chinese medicine, the treatment of osteoarthritis has showcased a unique cultural heritage and possesses a profound historical significance.

Compared to Western medical treatments, traditional Chinese medical therapies, including herbal medicine, acupuncture, and massage, have achieved both safety and positive clinical outcomes in the prevention and treatment of knee osteoarthritis.^12^^,^^19^^,^^20^ Acupuncture, in particular, is noted for its precise efficacy, convenience, cost-effectiveness, and lack of toxic side effects.^21^ Except for the traditional acupuncture method, warm acupuncture and electroacupuncture are frequently used, along with fire acupuncture, acupoint injection, and small needle knife therapy.^22^ The current aims to compare the clinical efficacy of four different acupuncture therapies ‒ traditional acupuncture, electroacupuncture, warm acupuncture, and fire acupuncture ‒ for knee osteoarthritis. The data showed that all four treatment strategies could improve the quality of life of the patients. But the effects of electroacupuncture were stronger than the other three treatments, which had improving effects on physical health, physical role functioning, bodily pain, vitality, social functioning, general health, and mental health of patients. The effects of warm acupuncture and fire acupuncture were weaker than electroacupuncture, but were stronger than traditional acupuncture. Similar results were also observed in the assessment of knee physical function by the WOMAC assessment form and detection of inflammatory indicators, with electroacupuncture showing better treatment effects than the other three treatments. However, the effects of different acupuncture strategies on detailed items might be different, the efficacy in treating knee osteoarthritis was comparable between different treatment strategies.

As a holistic therapy, acupuncture exerts a bidirectional regulatory effect on immune function, enhancing low immunity and suppressing excessive immune responses, which is closely related to the neuroendocrine-immune network system.^23^ It triggers the release of neurotransmitters such as acetylcholine and substances like enkephalins through the central descending pathway and the autonomic nervous system, regulating immune organs or lymphocyte surface receptors. Additionally, acupuncture regulates the endocrine system function, prompting the pituitary gland to release hormones such as adrenocorticotropic hormone and growth hormone, which modulate immune function. For instance, acupuncture can stimulate acupoints around the patient's knee joint, promoting the release of endogenous opioid peptides and other analgesic substances, thereby raising the pain threshold and achieving analgesic effects.^24^ Knee osteoarthritis is an inflammatory process involving various cytokines and inflammatory factors, particularly tumor necrosis factor, nitric oxide, interleukins, and prostaglandins. It is shown that acupuncture can reduce the concentrations of these substances in the serum, thereby exerting its anti-inflammatory effects and slowing cartilage degeneration. Additionally, the better treatment outcome of electroacupuncture might also be attributed to its inhibitory effects on the activities of these inflammatory factors.^25^ Compared with the other three treatment strategies, electroacupuncture involves applying pulse electrical stimulation to acupuncture points, combining both needle and electrical stimuli, which can enhance the efficacy of treatment for certain diseases.^26^ Moreover, this method allows for more precise control of stimulation parameters. Thus, in the current study, this approach is particularly effective in reducing joint and musculoskeletal pain associated with knee osteoarthritis, making its analgesic effects more pronounced. Regarding warm acupuncture and fire acupuncture, both methods exhibit the dual effects of acupuncture and moxibustion. Acupuncture can expel pathogens, while moxibustion and the thermal stimulation from fire can warm patients' bodies. The organic combination of these techniques achieves the effects of relieving pain.

Collectively, the current study compared the efficacy of four different acupuncture strategies in handling knee osteoarthritis, and found that all the strategies could improve the physical function and reduce pain in the knee, thereby improving the life quality of patients. Of the four strategies, electroacupuncture showed the strongest effects. However, the current study could not specify the exact time when each patient's pain was effectively relieved. For instance, after one course of treatment, the pain indices for the fire acupuncture group and electroacupuncture group were recorded, along with the pain scores for all four groups. While statistical analysis was performed on this data, the differences between the groups were not statistically significant. Additionally, the study failed to perform a long-term follow-up of the patients. Therefore, future studies with a larger sample size and multiple centers should be performed to verify the conclusion of the current study.

## Ethics approval and consent to participate

All patients were required to voluntarily agree to the use of their clinicopathological information in future analyses, such as in the current study and gave a written informed consent. The current study has been approved by the Ethics Committee of JiuJiang NO.1 People's Hospital, and was performed in accordance with the Declaration of Helsinki and STROBE Statement.

## Consent for publication

All patients were required to voluntarily agree to the use of their clinicopathological information in future analyses, such as in the current study, and gave a written informed consent.

## Clinical trial number

Not applicable.

## Availability of data and material

Data will be provided by the corresponding author on reasonable request.

## Funding

Not applicable.

## CRediT authorship contribution statement

**Jun Li:** Conceptualization, Data curation, Formal analysis, Writing – original draft. **Di Wu:** Data curation, Formal analysis. **Xueli Chen:** Data curation, Formal analysis. **Guanhua Li:** Conceptualization, Writing – review & editing.

## Conflicts of interest

The authors declare no conflicts of interest.
